# Multiple Hits, Including Oxidative Stress, as Pathogenesis and Treatment Target in Non-Alcoholic Steatohepatitis (NASH)

**DOI:** 10.3390/ijms141020704

**Published:** 2013-10-15

**Authors:** Akinobu Takaki, Daisuke Kawai, Kazuhide Yamamoto

**Affiliations:** Department of Gastroenterology and Hepatology, Okayama University Graduate School of Medicine, Dentistry and Pharmaceutical Sciences, 2-5-1 Shikata-cho, Kita-ku, Okayama City, Okayama 700-8558, Japan; E-Mails: daicawai@yahoo.co.jp (D.K.); kazuhide@md.okayama-u.ac.jp (K.Y.)

**Keywords:** molecular hydrogen, non-alcoholic steatohepatitis, oxidative stress

## Abstract

Multiple parallel hits, including genetic differences, insulin resistance and intestinal microbiota, account for the progression of non-alcoholic steatohepatitis (NASH). Multiple hits induce adipokine secretion, endoplasmic reticulum (ER) and oxidative stress at the cellular level that subsequently induce hepatic steatosis, inflammation and fibrosis, among which oxidative stress is considered a key contributor to progression from simple fatty liver to NASH. Although several clinical trials have shown that anti-oxidative therapy can effectively control hepatitis activities in the short term, the long-term effect remains obscure. Several trials of long-term anti-oxidant protocols aimed at treating cerebrovascular diseases or cancer development have failed to produce a benefit. This might be explained by the non-selective anti-oxidative properties of these drugs. Molecular hydrogen is an effective antioxidant that reduces only cytotoxic reactive oxygen species (ROS) and several diseases associated with oxidative stress are sensitive to hydrogen. The progress of NASH to hepatocellular carcinoma can be controlled using hydrogen-rich water. Thus, targeting mitochondrial oxidative stress might be a good candidate for NASH treatment. Long term clinical intervention is needed to control this complex lifestyle-related disease.

## 1. Introduction

Non-alcoholic fatty liver disease (NAFLD) is a common cause of chronic liver disease and a major indicator of metabolic syndrome that is becoming increasingly prevalent [1]. Non-alcoholic steatohepatitis (NASH) is a more severe form of NAFLD that is broadly defined by the presence of steatosis with inflammation and progressive fibrosis [2,3], ultimately leading to cirrhosis and hepatocellular carcinoma (HCC) [4–7]. A subset of patients with NAFLD develops NASH through poorly understood mechanisms.

**Figure 1:**
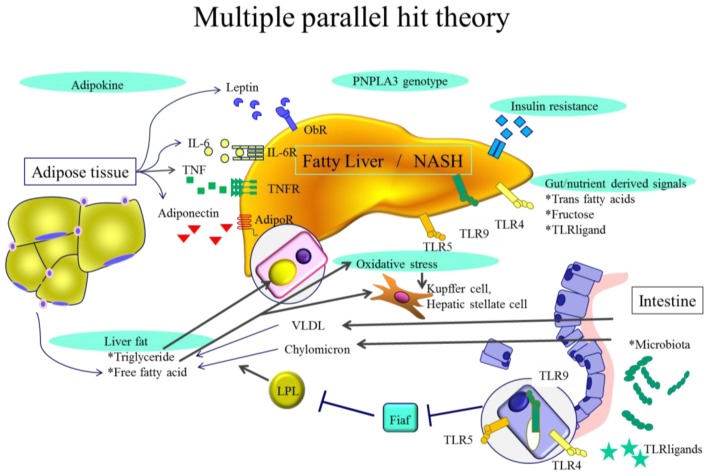
Multiple parallel hit theory. Genome-wide association studies have confirmed importance of *patatin-like phospholipase 3* (*PNPLA3*) gene polymorphism in NAFLD. This genetic polymorphism can differentiate simple steatosis with or without minimal inflammation and fibrosis progressing to NASH. In some instances, inflammation could precede steatosis and anti-tumor necrosis factor (TNF)-α antibody improves steatosis in ob/ob mice. Obesity and diabetes induce insulin resistance, adipocyte proliferation and changes in intestinal flora. Adipokines such as IL-6 and TNF-α produced by adipocytes affect hepatocyte fat content and liver inflammatory environment. Gut-derived signals could be affected by ingested trans fatty acids, fructose, or TLR ligands. Ingested free fatty acids and free cholesterol induce ER stress and oxidative stress resulting in hepatic inflammation and fibrogenesis.

The development of NASH has been considered a “two hit” process [8]. The first hit is the development of hepatic steatosis and the second hit includes cellular stresses such as oxidative stress, apoptosis and gut-derived lipopolysaccharide (LPS). However, a *patatin-like phospholipase 3* (*PNPLA3*) gene polymorphism also plays a key role in the development of NASH. Although fatty liver is usually non-progressive, it can progress in patients harboring the risk allele of the *PNPLA3* gene. Thus, the development of NASH including this genetic polymorphism is now regarded as a “multiple hit” process (Figure 1).

Many sources of cellular stresses, including oxidative stress, apoptosis and gut-derived LPS, trigger an inflammatory response and progressive liver damage [9]. Oxidative stress appears to be responsible for initiating necroinflammation, and reactive oxygen species (ROS) that are generated during free fatty acid metabolism in microsomes, peroxisomes and mitochondria comprise an established source of oxidative stress [10]. Mitochondria are the most important cellular source of ROS, and mitochondrial dysfunction might therefore play a central role in the pathological mechanisms of NASH. Although the mechanisms of mitochondrial dysfunction are not clearly understood, emerging data suggest that ROS, lipid peroxidation products and tumor necrosis factor-α (TNF-α) are involved in the second hit, which induces the progression of simple steatosis to NASH. Furthermore, ROS induce the directional migration of resident hepatic pro-fibrogenic cells, resulting in liver fibrosis [11].

Several studies have suggested that antioxidants such as vitamin E along with 1-aminobenzotriazole (ABT) and thiazolidinediones (insulin sensitizers) confer benefits upon patients with NAFLD or NASH [12–15]. In particular, the thiazolidinedione pioglitazone helps to improve insulin sensitivity, steatosis and inflammation. However, the improvement is not histologically clear [12]. Furthermore, most clinical studies of atherosclerotic diseases with dietary antioxidants have not generated clear results, partly because of the non-selective effects of these anti-oxidative drugs and difficulties associated with cytosolic distribution [16].

Molecular hydrogen has therapeutic value as an antioxidant through its ability to reduce one of the cytotoxic ROS hydroxyl radicals, but not superoxide, hydrogen peroxide or nitric oxide. Hydrogen is distributed into cytosol without any specific receptors or hydrophilicity [17]. Inhaled hydrogen gas (~4% H_2_ in air) can reduce infarct size in rat models of focal cerebral and myocardial ischemia reperfusion injury [18]. Drinking water containing therapeutic doses of hydrogen (hydrogen-rich water; HW) represents an alternative model for delivering molecular hydrogen to treat ROS-induced pathologies [19]. We recently showed that HW can counter oxidative stress in mouse models of NASH.

Here, we review the importance of oxidative stress in the pathogenesis of NASH and the effect of anti-oxidants including HW.

## 2. NASH Pathogenesis—General Characteristics

The pathogenesis of NASH is unclear. The two-hit theory that was suggested 15 years ago indicated that the first hit is hepatic steatosis and the second is caused by gut-derived endotoxins, oxidative stress or proinflammatory cytokines [20]. The concept of multiple parallel hits has recently been considered [21]. Inflammation can occasionally precede steatosis and patients with NASH can present without much steatosis, suggesting that inflammation can occur first [22]. Anti-tumor necrosis (TNF)-α antibody improves steatosis in ob/ob mice [23]. These results suggest that primary inflammation can induce secondary steatosis. Additionally, simple steatosis and NASH are considered separate diseases, each with a different pathogenesis.

### 2.1. Genetic Background

Genome-wide association studies (GWAS) have uncovered many disease- or treatment-susceptible genes. Several GWAS of various races have confirmed the importance of *PNPLA3* gene polymorphisms in NAFLD [24]. This genetic polymorphism differentiates between simple steatosis with or without minimal inflammation and fibrosis that progresses to NASH [25,26]. Patients with the NASH-sensitive single nucleotide polymorphism (SNP) rs738409 G/G genotype might progress not only to simple steatosis but also to NASH, probably under the same types of metabolic stimulation. The function of PNPLA3 is not well known since mice deficient in PNPLA3 develop neither fatty liver nor liver injury. However, the overexpression of sterol-regulated binding protein 1c (SREBP-1c) results in its binding to the transcription start site of the mouse *PNPLA3* gene and additional *PNPLA3* knockdown can decrease the intracellular triglyceride content in primary hepatocytes [27]. Thus, *PNPLA3* might function as a downstream target gene of SREBP-1c to mediate SREBP-1c stimulation of lipid accumulation. This genetic characteristic might be the “first hit” and subsequent hits might affect disease progression.

### 2.2. Visceral Obesity

Obesity is a growing global epidemic among adults and children that is associated with many diseases such as hypertension, diabetes mellitus, hyperlipidemia and NAFLD. Obesity, hypertriglyceridemia, and hypertension are predictive risk factors for NAFLD [28]. Visceral fat accumulation in obesity correlates with various organ pathologies including cerebrovascular diseases, cancer and NASH. Visceral fat accumulation is regarded as a significant risk factor for the development of NAFLD and NASH. A study from Japan has found that the severity of hepatic steatosis determined by ultrasound positively correlates with visceral fat accumulation and insulin resistance in both obese and non-obese individuals, suggesting that hepatic steatosis is influenced by visceral fat accumulation regardless of obesity [29]. Visceral obesity has become quite common even among children world-wide over the past decade, thanks to a shift towards Western style diets that are high in calories, fat and fructose [30].

### 2.3. Insulin Resistance

Insulin resistance is an independent risk factor for NAFLD severity [29]. Adipose and hepatic insulin resistance progressively increases across NAFLD stages even in non-obese, non-diabetic and normolipidemic patients. The oral glucose tolerant test (OGTT) shows impaired pancreatic β-cell function in patients with NASH but not in those with simple steatosis [31]. Also, a more atherogenic postprandial lipoprotein profile indicates systemic insulin resistance and hepatic free fatty acid (FFA) accumulation.

### 2.4. Hepatic Steatosis

Fatty liver is the basic feature of NAFLD and NASH. Triglycerides are the main types of lipids stored in the liver of patients with NAFLD. The toxic lipids in NASH and the non-toxic lipids in simple steatosis might be different [32]. Diacylglycerol acyltransferase 2 (DGAT2) catalyzes the final step in hepatocyte triglyceride biosynthesis. Hepatic steatosis and the dietary triglyceride contents induced in a model of obese-simple fatty liver are reduced by DGAT2 antisense oligonucleotides in a manner that does not correlate with changes in body weight, adiposity or insulin sensitivity [33]. However, a DGAT2 antisense oligonucleotide increased levels of hepatic free fatty acids, lipid oxidant stress, lobular necroinflammation and fibrosis in mice fed with a methionine choline-deficient (MCD) diet that generates inflammation and fibrosis with hepatic steatosis, whereas hepatic triglyceride content decreased [32]. These results suggest that the pathogenesis and treatment of steatosis in simple fatty liver and in NASH are different. Human genetic variability analysis of a lifestyle intervention has shown that the *DGAT2* gene polymorphism is related to a decrease in liver fat, while changes in insulin resistance do not correlate [34]. Since insulin resistance is the key marker for NASH, the *DGAT2* gene polymorphism might only be associated with non-progressive fatty liver.

## 3. NASH Pathogenesis—Cellular Levels

Since the significance of apparently similar fat droplets in simple fatty liver and NASH hepatocytes differ in DGAT2 knockdown experiments, analyzing the molecular pathogenesis of NASH at the cellular level is quite important.

### 3.1. Adipokines

Adipokines are multifunctional secreted factors that are primarily considered to be derived from adipose tissue. Such tissue has been regarded simply as a means of storing energy, whereas it is now considered to be a complex organ that is involved in the control of many processes including metabolic, immunological and inflammatory responses. Adiponectin is the most abundant and adipose tissue-specific adipokine. Mature adipocytes mainly produce adiponectin in white adipose tissue and expression and secretion levels increase during adipocyte differentiation. Levels of adiponectin are significantly higher in females than in males, in whom serum androgens become more evident during puberty [35]. Adiponectin levels inversely correlate with visceral obesity and insulin resistance and weight loss is an inducer of adiponectin synthesis. Proinflammatory adipokines such as TNF-α or IL-6 suppress adiponectin, which has anti-inflammatory, anti-atherogenic and anti-diabetic properties [36]. Adipose tissue is also the main producer of the adipokine leptin and its levels directly correlate with body fat mass and adipocyte size [37]. Leptin production is mainly regulated by food intake, and hormones related to eating such as insulin increase leptin secretion and vice versa. Leptin is also regulated by sex hormones, because testosterone inhibits leptin production whereas ovarian sex steroids increase it, resulting in higher levels in females. Proinflammatory endotoxin, IL-1 and TNF-α increase the secretion of leptin, which has central and peripheral effects. Leptin acts on hypothalamic cells, inhibits anabolic pathways, activates catabolic pathways, inhibits appetite and stimulates energy expenditure. Peripheral leptin increases basal metabolism, regulates pancreatic cell functions and insulin secretion, and affects T cell generation and the differentiation of T helper 1 cells in lymph nodes. Leptin-deficient (ob/ob) mice and leptin receptor-deficient (db/db) mice are severely obese and have increased pituitary and adrenal hormone production, hyperglycemia, elevated insulin and decreased immune function.

Several groups have rather found that serum adiponectin levels are controversially lower in NAFLD than in NASH or are the same [38–41]. A meta-analysis of 27 studies of 698 controls and 1545 patients with NAFLD found that serum adiponectin levels are low in NAFLD and much lower in NASH [36]. Since adiponectin and leptin exert antagonistic effects on liver fibrogenesis and inflammation, the ratio of adiponectin to leptin might be a better marker with which to distinguish NASH from NAFLD. Levels of adiponectin receptor II are decreased in human liver biopsy specimens and in mouse models of NASH [42,43]. However, since contradictory results have suggested that lower serum adiponectin induce high expression of hepatic adiponectin receptor II as a compensatory response, the function of these novel adipokines and receptors requires further investigation [44,45].

### 3.2. Endotoxin and Gut Derived Signals

Gut microbiota play many roles in NAFLD and NASH, hepatocellular carcinoma, cardiac functions, vascular atherosclerosis, diabetes and other conditions. Endotoxin or LPS produced by gut microbiota could be delivered to the liver via the portal vein, which raises the question of why such toxic materials flow into the portal vein through the intestinal barrier. Patients with biopsy-proven NAFLD have increased intestinal permeability with disrupted intercellular tight junctions in the intestine [46]. These abnormalities are related to increased bacterial overgrowth in the small intestine. Murine NAFLD models of bacterial overgrowth develop compositional changes and increased intestinal permeability with a concurrent reduction in the expression of tight junction proteins [47]. Plasma endotoxin levels are significantly higher in patients with NAFLD and in murine NASH models [46,48]. A high-fat diet could increase LPS concentrations two- to three-fold [49]. Proinflammatory inflammasomes induce inflammation in the liver of patients with NAFLD, but an inflammasome-deficient model developed exacerbated hepatic steatosis and inflammation through the influx of TLR4 and TLR9 agonists into the portal vein [50]. The microbiota of these inflammasome-deficient mice differed from those of wild-type mice with NASH. Furthermore, co-housing inflammasome-deficient and wild-type mice resulted in intestinal inflammation and exacerbated hepatic steatosis in the wild-type mice. This finding suggested that altered microbiota in inflammasome-deficient mice could be transferred to healthy mice resulting in intestinal inflammation, increased permeability and NAFLD. The gut and oral periodontal status correlates with the progression of liver disease [51]. Treating periodontitis could improve transaminases in NAFLD and, in fact, several probiotics that control gut microbiota improve NAFLD [52–54]. Studies using models of hepatocarcinogenesis have found that a high-fat diet increases levels of deoxycholic acid, a gut bacterial metabolite that damages DNA and exacerbates hepatocarcinogenesis [55]. Antibiotics could abrogate these effects. Gut microbiota affect not only NAFLD, but also obesity-related hepatocarcinogenesis.

### 3.3. Toll-Like Receptors (TLRs)

Toll-like receptors are sensors of microbial and endogenous danger signals that are expressed and activated in innate immune cells and in liver parenchymal cells and they contribute to the progression of NASH. Gut microbiota might release pathogen- or damage-associated molecular patterns (PAMPs or DAMPs), which are TLR ligands following the activation of downstream signals. Among 10 TLRs (TLR1-10) identified in humans and 13 (TLR1-9, 11–13) determined in mammals, TLR2, TLR4, and TLR9 seem to be involved in the pathogenesis of NASH. Toll-like receptor 2 is a receptor for multiple glycolipids or lipoproteins in bacteria adhering to the cell surface of monocytes, myeloid dendritic cells or mast cells. Toll-like receptor 4 is a LPS ligand located on the surfaces of monocytes, myeloid dendritic cells, mast cells, B cells and intestinal epithelium. This receptor ligated with representative pathogens of gut microbiota has been studied in detail. Levels of free cholesterol (but not of cholesterol ester) are increased in hepatic stellate cells (HSC) in NAFLD resulting in increased TLR4 protein levels and fibrogenic HSC [56]. Kupffer cells are phagocytes of various cellular, viral, or bacterial components that are sources of hepatic pro-inflammatory and pro-fibrogenic cytokines. Cholesterol phagocytosed to Kupffer cells can induce their activation along with TLR4 upregulation [57]. Toll-like receptor 9 is located on endoplasmic reticulum (ER) or endosomes of plasmacytoid dendritic cells or B cells and is regarded as a ligand for unmethylated CpG DNA particles that might be released from bacteria. These molecules have been analyzed in several NAFLD and NASH models. Free cholesterol can accumulate in fibrogenic hepatic stellate cells, resulting in an increase in TLR4 through suppressing the endosomal-lysosomal degradation pathway of TLR4. The increased expression of TLR4 sensitizes cells to TGF-β induced activation [56]. The results of TLR studies in different NASH models notably vary. So far, mouse NASH models can represent only a portion of the pathogenesis. For example, the choline-deficient amino acid-defined (CDAA) diet model mouse develops steatosis with relatively mild hepatitis or fibrosis with obesity, whereas the methionine and choline deficient (MCD) diet model mouse develops steatosis with severe hepatitis and fibrosis without obesity. The CDAA diet improved NASH outcomes, whereas the MCDD diet worsened NASH outcomes in TLR2 knockout mice [58,59]. This discrepancy might be true since NASH itself is also heterogeneous and complex. TLR4 and TLR9 agonists might flow into the portal veins of inflammasome-deficient MCDD mouse models and thus exacerbate NASH [50]. Inflammasomes are multiprotein complexes composed of nucleotide-binding domain and leucine-rich repeat protein 3 (NLRP3), apoptosis-associated speck-like protein containing CARD (ASC) and procaspase 1 that are DAMP or PAMP sensors. Inflammasome activation leads to the processing and secretion of proinflammatory cytokines IL1β and IL-18, whereas knockdown results in MCD NASH exacerbation. These perplexing findings indicate that the intestinal DAMP or PAMP barrier function that is disrupted in inflammasome knockout mice overcomes the anti-inflammatory effect in the liver and TLR4 and TLR9 ligands outflow into the portal vein where they stimulate NASH progression. Toll-like receptor 5 is the ligand for microbial flagellin. Mice that are deficient inTLR-5 develop spontaneous colitis, profound metabolic syndrome including hyperphagia, hypertension and insulin resistance brought about through intestinal microbiota transport [60,61]. However, another study found no such pathologies in TLR5-deficient mice [62]. The authors concluded that this discrepancy might have been induced by their animal facilities, an indication that might be important to consider for all such molecular functions.

### 3.4. Endoplasmic Reticulum (ER) Stress

Toxic lipids such as free fatty acids, diacylglyceride, phospholipids and free cholesterol activate several cellular stress pathways [63]. One epicenter for these stress responses is the ER, a membranous network that functions in the synthesis and assembly of secretory and membrane proteins to achieve their proper conformation. Proteins that are related to lipid metabolism are enriched whereas protein synthesis and transport functions are downregulated in obese hepatic ER [64]. The maintenance of ER function requires high concentrations of intra-ER Ca^2+^, which is actively controlled by sarco (endo) plasmic reticulum Ca^2+^-ATPase (SERCA). Free cholesterol accumulation triggers ER stress by altering the critical free cholesterol-to-phospholipid ratio of the ER membrane, which is needed to maintain its fluidity. Among the ER enzymes, SERCA ATPase is particularly sensitive to ER membrane cholesterol contents that can inhibit SERCA conformational changes and activity. Such changes induce a decrease in physiologically high intra-ER Ca^2+^ concentrations that result in impaired ER function known as ER stress. Such stress is one of the most important factors for disease progression in NASH along with hepatocyte apoptosis and hepatic stellate cell (HSC) or Kupffer cell activation.

The chaperone response is blunted in obese mice as a result of dysfunctional X-Box binding protein 1 (XBP1), which is a master regulator of ER folding capacity [65]. Levels of liver-specific SERCA2b are reduced in obese mice with increased ER stress. Increasing SERCA2b levels reduces ER stress in the liver and increases glucose tolerance [66]. The SERCA inhibitor thapsigargin induces an increase in cytosolic calcium contents with a decrease in ER calcium resulting in mitochondrial cytochrome C release and the apoptosis of cultured primary rat hepatocytes [67]. Kupffer cells function as antigen presenting cells by displaying major histocompatibility complex (MHC) peptides on the cell surface. These peptides are processed by proteasomal degradation in the cytosol and then translocated to the ER, where they undergo *N*-terminal trimming and loading onto MHC for export to the cell surface. The ER also contains inflammatory cytokine-inducible factors such as stimulator of interferon genes (STING) that induce type I interferon genes upon release [68]. Endoplasmic reticulum stress induces the activation of such MHC-related and non-related pro-inflammatory responses. These results suggest that diseases could be treated with anti-ER stress agents as noted in the above model mice. However, ER stress also sensitizes activated, but not quiescent HSC to apoptosis, resulting in the resolution of fibrosis [69]. Non-selective anti-ER stress treatment might be ineffective and treatment targeted towards ER stress should be designed to focus on the specific status of ER conditions.

### 3.5. Oxidative Stress

#### 3.5.1. Oxidative Stress in Hepatocytes

**Figure 2:**
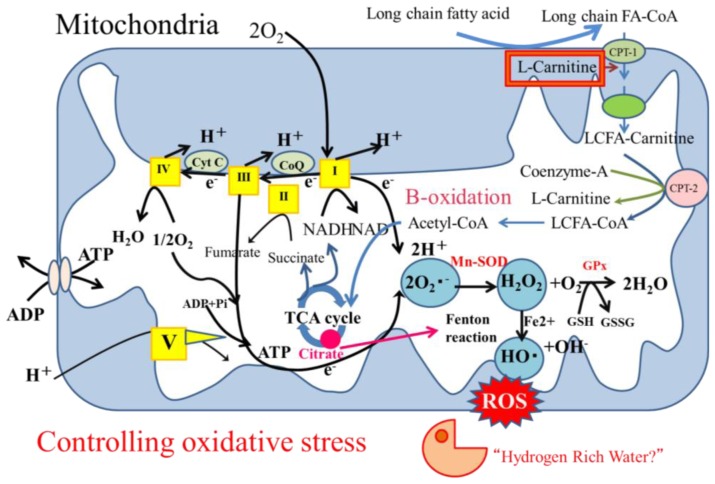
Mitochondria as producers of oxidative stress. High levels of plasma free fatty acids allow upregulation of hepatic free fatty acids. Long-chain fatty acids taken up into mitochondria as complexes with l-carnitine are then transferred to β-oxidation pathway. Under oxidative stress, oxidative reactions convert oxidized cofactors (NAD+ and FAD) into reduced cofactors (NADH and FADH2) and deliver electrons to respiratory chain. Imbalance between increased delivery of electrons to respiratory chain and their decreased outflow from this chain causes electrons and ROS products to accumulate. Anti-oxidant defenses such as superoxide dismutase (SOD), glutathione peroxidase (GPx) or catalase can metabolize hydrogen peroxide to non-toxic H_2_O. However, the Fenton and/or Haber-Weiss reactions generate highly reactive toxic ROS, hydrogen peroxide. Hydrogen as selective cytotoxic ROS scavenger and l-carnitine as mitochondrial function supporting factor might be good candidates for controlling mitochondrial oxidative stress.

Oxidative stress is involved in the mechanisms of aging, carcinogenesis and atherosclerotic progression. Excessive oxidative stress induced by mitochondrial, peroxisomal and microsomal reactive oxygen species (ROS) in NASH results in apoptosis as well as damage to nuclear and mitochondrial DNA. Limited antioxidant defenses contribute to the processes of both NASH and hepatocarcinogenesis [70,71]. Physiologically low levels of ROS are involved in necessary vital cellular processes indicating that the balance of oxidative anti-oxidative responses is important [72]. An imbalance in the mitochondrial respiratory chain is the main source of ROS, superoxide, hydrogen peroxide, and hydroxyl radicals. Acute oxidative stress can be induced by several types of organ stress such as inflammation, organ infarction, shock and reperfusion injury. Under oxidative stress, oxidative reactions such as β-oxidation, tricarboxylic acid (TCA) cycle turn oxidized (NAD+ and FAD) into reduced (NADH and FADH2) cofactors (Figure 2). Subsequent oxidation of these reduced cofactors results in the delivery of electrons to the final acceptor molecular oxygen through the mitochondrial respiratory chain, namely the electron transport chain (ETC; complexes I, III, and IV). Under physiological conditions, most reactive, incompletely reduced forms of oxygen, such as superoxide, are detoxified into water by various anti-oxidant defenses and repair enzymes to maintain a relatively low steady state of oxidants.

The mitochondrial capacity to control the oxidative balance will collapse under continuous oxidative stress. Excess superoxide could be generated within injured mitochondria through electron leakage and a resulting excess of superoxide would be converted to hydrogen peroxide by superoxide dismutase (SOD). Glutathione peroxidase (GPx) or catalase can metabolize hydrogen peroxide to non-toxic H_2_O, but the Fenton and/or Haber-Weiss reactions generate highly reactive toxic ROS, hydrogen peroxide. Such oxidative stress produced by Fenton reactions is mediated by iron. Although its role in NASH is not fully understood, levels of iron are elevated in NASH, which is an inducer of oxidative stress and reduced iron levels resulted in fair outcomes for patients with chronic liver diseases [73]. Elevated plasma citrate levels in NAFLD promotes iron mediated hydroxyl radical formation *in vitro* [74]. Excess fatty acid and glucose results in the elevation of pyruvate and acetyl-CoA that subsequently increases the formation of citrate, which induces iron related oxidative stress. The reactivity of ROS dictates toxicity and high reactivity results in a short life and limited diffusion (half life: superoxide and hydrogen peroxide 10^−6^ and 10^−9^ sec, respectively) [72]. However, ROS can attack polyunsaturated fatty acids (PUFA) and initiate lipid peroxidation resulting in the formation of aldehyde by-products such as 4-hydroxy-2-nonenal (4-HNE) or malondialdehyde (MDA) [75]. These 4-HNE and MDA have longer half-lives than ROS and they can diffuse into other sites and thus spread oxidative stress. Mitochondrial free fatty acid oxidation is not inhibited until respiration is severely impaired, resulting in accelerated ROS production until mitochondria are lost. Thus, mitochondria comprise the main source of oxidative stress, ER stress can induce superoxide and hydrogen peroxide with monooxygenases such as cytochrome P450 and peroxisomes can induce cytosolic hydrogen peroxide associated with fatty acid oxidation [76,77].

Mitochondrial antioxidant defenses are not powerful enough to control continuous oxidative stress such as that imposed by long chain free fatty acids and this results in oxidative damage to the ETC complexes and mitochondrial DNA [78,79]. The production of ROS in the inner membrane of mitochondria easily attacks proximal mitochondrial DNA. However, DNA repair mechanisms are thought to play a central role in preventing the accumulation of DNA mutations and in the maintenance of DNA stability [80]. Although both nuclear and mitochondrial DNA repair pathways exist, mitochondria are deficient in several of them [80]. Estrogen can recover one pathway of mitochondrial DNA repair, namely the base excision repair (BER) pathway that becomes impaired in aged female rats, indicating that estrogen confers benefits on DNA pathways in females [81]. Because of these mitochondrial DNA deficiencies in guarding against stress reactions, mitochondrial DNA could be easily broken or mutated by oxidative stress [82]. The accumulation of mitochondrial DNA mutations and damage results in a dysfunction of the ETC and leads to increased ROS as well as subsequent additional DNA damage. These oxidative stress chain reactions produce large amounts of ROS and target other mitochondria or organelles and induce cellular apoptosis.

The mitochondrial proliferation and differentiation program might be impaired in NASH. The most important regulator of mitochondrial biogenesis is the transcription coactivator peroxisome proliferator-activated receptor (PPAR)-γ-coactivator-1α (PGC-1α) [83] that coordinates the large number of genes required for mitochondrial biogenesis. The activity of PGC-1α is impaired in the fatty liver, which results in a decrease in mitochondrial biogenesis [84]. Decreases in mitochondrial DNA and mitochondrial DNA-encoded polypeptides are representative findings in NASH, whereas the mitochondrial DNA content is increased in simple fatty liver [85]. The complementary activation of mitochondrial DNA in simple fatty liver might help to protect the liver from inflammation and fibrosis, whereas a decrease in NASH induces progressive inflammation and fibrosis.

#### 3.5.2. Oxidative Stress Affects outside Hepatocytes

Among the non-parenchymal cells that comprise almost 40% of all liver cells, Kupffer cells and HSC play significant roles in the progression of chronic liver inflammation and fibrosis progression [75]. Excess fatty acid accumulation in hepatocytes induces oxidative stress from not only mitochondria but also peroxisomes or microsomes. These cytotoxic ROS and lipid peroxidation products can diffuse into the extracellular space affecting Kupffer cells and HSC. These cellular oxidative stresses from hepatocytes and the direct uptake of free fatty acids or free cholesterol in Kupffer cells induce the activation of nuclear-factor κB, which induces the synthesis of TNF-α and several proinflammatory cytokines such as IL-6 or IL-8 [86]. Kupffer cells in patients with NASH produce TGF-β resulting in HSCs acquiring a fibrogenic myofibroblast-like phenotype.

Exposing primary HSC or HSC cell lines to hydrogen peroxide leads to an increase in the gene expression of ER chaperone BIP binding transmembrane proteins such as inositol requiring enzyme 1 (IRE1a), or activating transcription factor 4 (ATF4). These ER stresses in HSCs result in increased autophagy and HSC activation to fibrogenic status [87]. Among the most characteristic features of HSC activation is the loss of cytoplasmic lipid droplets, which are composed of retinyl esters and triglycerides [88]. Autophagy is present in all cell types and it is up-regulated as an adaptive response under cellular stress to generate intracellular nutrients and energy. Autophagy is up-regulated in activated HSC of mice with liver damage. However, cytoplasmic lipid droplets are maintained and stay quiescent in autophagy-defective HSCs, representing oxidative stress-induced ER stress and autophagy as a key event in HSC activation [89].

## 4. Treatment for NASH

### 4.1. General Aspects

Since NAFLD and NASH have emerged as lifestyle-associated diseases, lifestyle intervention is an important approach to their treatment. Healthy and Western-style diets differentiate the risk for NAFLD progression [90]. Even a one-year intensive lifestyle intervention that comprised dietary modifications and physical activity improved waist circumference, visceral abdominal fat, blood pressure, insulin resistance and hepatic fat content in obese patients [91]. Western-style diets, especially those that are rich in trans-fatty acids, are powerful inducers of obesity and NAFLD needs to be avoided [92]. However, to maintain compliance with such therapeutic measures as restrained food consumption is mentally challenging and the rebound rate is high [93]. Such patients require pharmacological therapies.

Because the general characteristics of NASH comprise obesity and insulin resistance, anti-insulin resistance treatment has played significant roles. From this viewpoint, the ability of insulin sensitizing anti-diabetic drugs to treat NASH has been analyzed. Among these, the PPAR-α agonist pioglitazone and metformin have clinically improved NASH. However, neither of these drugs could improve liver histology. Metformin induced weight loss, whereas pioglitazone induced weight gain. Metformin has brought about histological improvements in the non-diabetic NASH mouse model [94]. Human trials are usually too short to generate histological improvements. These anti-diabetic insulin sensitizing drugs also have anti-oxidative effects. Glucagon like peptide-1 (GLP-1) and gastric inhibitory polypeptide (GIP) are both incretins, which are a group of gastrointestinal hormones that cause increased insulin release from pancreatic beta cells, and they might be good targets for treating NASH. A GLP-1 receptor agonist analogue improved metabolic, biochemical and histopathological indices of NASH in mice via restoring hepatic lipid oxidation [95]. A GLP-1 receptor agonist in type 2 diabetic patients with NAFLD caused a reduction in intrahepatic lipid content that correlated with diabetic control [96]. Dipeptidylpeptidase-IV (DPP-IV) degrades GLP-1 and GIP and thus the inhibition of DPP-IV extends the half-life of endogenous GLP-1 and GIP, resulting in diabetic control. The long-term administration of a DPP-IV inhibitor has reduced liver fat content in animals with diet-induced hepatic steatosis and insulin resistance [97].

The representative antioxidant vitamin E improved the non-alcoholic fatty liver disease activity scores (NAS) for clinical and histological activity within two years but increased insulin resistance and plasma triglyceride levels [12]. However, the recovery of fibrosis progression was not proven [98]. Antioxidants had no effect on body weight, waist circumference and cholesterol metabolism. l-carnitine is a precursor of carnitine-palmitoyltransferase 1 (CPT-1), the rate-limiting enzyme for mitochondrial β-oxidation that affects mitochondrial function. Any deficiency in the mitochondrial carnitine-dependent transport system results in curtailed fatty acid oxidation. l-carnitine supplementation reduces TNF-α, liver function parameters, plasma glucose levels and histological scores [99]. Pentoxiphylline is a methylxanthine derivative that increases red blood cell flexibility, reduces blood viscosity and decreases platelet aggregation. In addition, pentoxiphylline suppresses TNF-α gene transcription and it is a hydroxyl and peroxyl radical scavenger that results in anti-oxidative effects. A randomized controlled trial (RCT) has proven that pentoxiphylline decreases free-radical-mediated lipid oxidation and improves clinical and histological NASH [100,101].

Lipid-lowering drugs such as statins can also improve ALT and radiological steatosis in hyperlipidemic patients with NAFLD, but histological improvements are not evident [102]. Ezetimibe is a Niemann-Pick C1-like protein inhibitor that can reduce the intestinal accumulation of free cholesterol. Ezetimibe has reduced histological NAS improvement in mice and in 10 patients with NASH, indicating a need for larger RCT [103,104].

Ursodeoxycholic acid (UDCA) is also reportedly effective in some instances. Several RCT have found improvements in ALT but not in liver histology even at high doses [105]. Combinations of UDCA and vitamin E have improved ALT and histological NAS scores [106].

Tumor necrosis factor (TNF)-α is one of the main cytokines involved in adipocyte-related inflammation including NASH. Powerful anti-TNF-α agents such as infliximab (a chimeric monoclonal antibody), adalimumab (a human monoclonal antibody) and etanercept (a fusion protein) have severe side effects such as tuberculosis that would render them unacceptable as therapy for NAFLD [107]. The anti-oxidative agent pentoxifylline described above also has an anti-TNF-α function that is partially involved in the favorable effects on NASH.

A pro-inflammatory intestinal microbiome has been identified in mice and in patients with NASH. Probiotics such as butyrate-producing agents reduce hepatic triglyceride contents and induce anti-oxidative enzymes that help to prevent the progression of NASH to hepatocellular carcinoma [53].

### 4.2. Hydrogen-Rich Water as a Candidate Treatment for NASH

#### 4.2.1. Hydrogen as an Antioxidant Treatment

Controversy surrounds antioxidant therapies because ROS have essential functions in living organisms. Balancing oxidative stress is the key issue, and bioavailability and bioaccessibility are needed to elicit an effective response to drugs. Antioxidants have effective chemical activities *in vitro*, however, many failures have been shown in proving *in vivo* fair effects [108]. Many cerebrovascular studies have investigated the effects of the representative antioxidant vitamin E. A meta-analysis of the effect of vitamin E on stroke revealed a 10% reduction in ischemic stroke accompanied by a 22% increase in hemorrhagic stroke. Antioxidants are likely to cause the progression of cancer [109]. Stem cell like cancer cells have powerful antioxidative properties that protect them from oxidative stress and thus prevent their apoptosis [110]. Oxidative stress upon normal cells might induce them to become cancer cells that are highly resistant to further oxidative stress. Inducing oxidative stress under these conditions is an approach to treat cancers that several trials are investigating [111]. However, this approach is likely to kill normal cells and induce new cancer development from normal cells. Thus, oxidative stress must be controlled according to clinical circumstances. The clinical findings of antioxidant therapies have not always been favorable [112]. This might be because mitochondria do not effectively take up antioxidants that would then interfere with the essential mechanisms of oxidative stress that protects cells from infection or other invasive cellular injury [113]. Ohsawa *et al.* [18] have found that molecular hydrogen has powerful antioxidant effects with unique features. In cultured cells H_2_ scavenges hydroxyl radicals but not superoxide or hydrogen peroxide and nitric oxide (NO). Levels of intracellular superoxide increased in cells cultured with the mitochondrial respiratory complex III inhibitor, antimycin A and then rapidly converted to hydrogen peroxide and hydroxyl radicals. Culture with H_2_ decreased levels of hydroxyl radicals but not those of superoxide, hydrogen peroxide or steady-state levels of NO in cells. Even nuclear levels of hydroxyl radicals were notably decreased. As a result, H_2_ prevented a decline in the mitochondrial membrane potential and the decrease in cellular ATP synthesis suggestive of effective antioxidants.

To defend cells against bacterial invasion, hydrogen peroxide is converted to hypochlorous acid by myeloperoxidase, indicating that oxidative stress is important for survival [114]. Additionally, NO functions as a neurotransmitter that is essential for blood vessel dilation and it protects against endothelial cell activation, suggesting that oxidative stress is important for survival [115]. Treatment with H_2_ reduces levels of hydroxyl radicals but not those of superoxide or hydrogen peroxide, which have physiological roles in cell survival.

Most hydrophilic compounds are retained at membranes and never reach the cytoplasm, whereas hydrophobic compounds such as vitamin E cannot penetrate biomembranes in the absence of specific carriers or receptors. In contrast, H_2_ can diffuse into cytoplasm and intracellular organelles such as mitochondria, the ER and the nucleus.

High concentrations of H_2_ are not cytotoxic. Breathing high concentrations of H_2_ in gas has been used to treat decompression sickness and arterial gas thrombosis after deep diving [116].

Several disease models have been created to determine the effects of molecular hydrogen (Table 1) [19,70,117–129]. Hydrogen can be ingested mainly by gas inhalation or drinking hydrogen-rich water. Hydrogen can be inhaled via hydrogen gas delivered through a ventilator with a face mask. Arterial blood levels of H_2_ increase depending upon the concentration of inhaled H_2_ gas. The diffusion of H_2_ gas has been monitored in the rat myocardium [118], in which the H_2_ concentration was increased by two thirds in the ischemic compared with the non-ischemic myocardium. Ischemic volume was decreased one day after middle cerebral artery occlusion in a rat model of cerebral infarction that had inhaled H_2_, the wider volume difference one week after occlusion between these and control rats indicated improvements in chronic ischemic stress [18]. The inhalation of H_2_ reduces inflammatory responses associated with ventilator-induced lung injury at local and systemic levels via its antioxidant, anti-inflammatory and anti-apoptotic effects [130].

Drinking hydrogen-rich water is a straightforward method of daily administration at outpatient clinics because up to 0.8 mM H_2_ (1.6 ppm, *w/v*) can be dissolved in water under atmospheric pressure. Glass or plastic containers are unsuitable for conserving H_2_ since it can penetrate rapidly, whereas aluminum bags can retain H_2_ for long periods [113]. Hydrogen-rich water can be prepared by dissolving hydrogen gas in water under high pressure or by the reaction of magnesium metal with water. The detection of H_2_ at μM levels in the liver and the fact that the H_2_ concentration peaks at five minutes after hydrogen-rich water reaches the stomach suggests that the liver is a good H_2_ target [122].

#### 4.2.2. Hydrogen as an Antioxidant Treatment Candidate for NASH

The effects of hydrogen on chemically induced liver damage have been studied in mouse models of liver damage induced by GalN/LPS, CCl_4_ and diethylnitrosamine (DEN) [125]. Hydrogen was given intraperitoneally every three hours after the administration of chemicals. Serum levels of TNF-α and IL-6 and transaminase levels decreased in mice with GalN/LPS-induced acute liver injury after H_2_ administration. Hepatic fibrogenesis markers such as collagen-α1 or α-smooth muscle actin (SMA) were reduced in model mice with CCl_4_-induced liver cirrhosis and proliferative activities of hepatocytes were reduced in mice with DEN induced hepatic tumorigenesis.

Kawai *et al.* have reported that drinking hydrogen-rich water has favorable effects in NASH models [70]. Plasma transaminase levels, histological NAS, hepatic TNF-α, IL-6 and fatty acid synthesis-related gene expression and the oxidative stress biomarker 8-OHdG were decreased in the livers of established MCD diet-induced NASH models administered with hydrogen-rich water or anti-oxidative pioglitazone. Although the decrease in hepatic cholesterol was smaller in the group given hydrogen-rich water, serum oxidative stress was reduced and antioxidant function was higher than that in the pioglitazone group. Another NASH mouse model was constructed to determine whether hydrogen affects hepatocarcinogenesis. The Stellic Research Institute created a mouse model of streptozotocin-induced NASH (STAM) that represents hepatocarcinogenesis within 16 weeks [131]. Hydrogen-rich water reduced the number and size of hepatocellular carcinoma lesions in this model compared with controls.

The consumption of hydrogen-rich water might effectively treat NASH by reducing hepatic oxidative stress, inflammation, and hepatocarcinogenesis. We emphasize that because the results of many investigations into the effects of antioxidants upon diseases associated with oxidative stress have been disappointing, hydrogen might be the same as those of previously discouraged agents. More basic and clinical understanding of this novel potential treatment option is required.

## 5. Conclusions

Multiple parallel hits derived from lifestyles are now believed to cause NASH. The pathogenesis of cellular damage is also heterogeneous, but oxidative stress seems to be one key factor that affects the progression of NASH. Several approaches to control this hidden phenomenon are often unsuccessful, because it is also essential for life. However, new approaches that target mitochondria such as l-carnitine, pentoxyphyllin, or molecular hydrogen have favorable effects. Optimal treatment protocols and combinations of these elements should be further investigated.

## Figures and Tables

**Table 1:** Effectiveness of hydrogen.

Targets	Hydrogen Administration	Reference Number
*Disease models*		

Cerebral ischemia reperfusion injury	Gas	[18]
Liver ischemia reperfusion injury	Gas	[111]
Myocardial ischemia reperfusion injury	Gas	[112]
Lung ischemia reperfusion injury	Gas	[113]
Parkinson’s disease	Drinking water	[114]
Atherosclerosis	Drinking water	[115]
Obese diabetes	Drinking water	[116]
Type1 diabetes	Drinking water and intraperitoneal injection	[117]
Diabetic retinopathy	Intraperitoneal injection	[118]
Chemically-induced liver injury	Intraperitoneal injection	[119]
NASH	Drinking water	[70]
Cisplatin-induced renal injury	Gas and drinking water	[120]
Post renal transplant rejection	Drinking water	[19]
Intestinal transplant rejection	Graft storage in hydrogen bubbled preservative	[121]

*Clinical trials*		

Glucose and lipid metabolism type 2 diabetes	Drinking water	[122]
Metabolic syndrome	Drinking water	[123]
